# Can CHATGPT provides reliable technical medical information about phimosis?

**DOI:** 10.1590/S1677-5538.IBJU.2024.9913

**Published:** 2024-05-31

**Authors:** Edson S. Salvador, Carla S. Santos, Vimael J. O. Holanda, Bruno M. Corrêa, Luciano A. Favorito

**Affiliations:** 1 Universidade do Estado do Rio de Janeiro Unidade de Pesquisa Urogenital Rio de Janeiro RJ Brasil Unidade de Pesquisa Urogenital, Universidade do Estado do Rio de Janeiro – UERJ, Rio de Janeiro, RJ, Brasil

## COMMENT

Phimosis is the most common clinical condition in pediatric urology. Physicians must be trained to answer patients and parent's questions about phimosis and circumcision and make clinical decision based on medical data and patient's medical context. Nowadays, a lot of medical and non-medical source of educational information are disponible for physicians, parents, and patients on internet, such as: Pubmed®, Google Scholar®, Instagram®, Youtube® and chatbots platforms. ChatGPT v3.5 ( 1 ) (OpenAI, California, 2022), is a chatbot platform that, according to own ChatGPT definition, is an "Artificial Intelligence system trained on diverse internet text and is capable of answering questions, providing information's and engaging in conversations." ( 1 ).

Patients and physicians use ChatGPT as a "search engine" to answer routine medical topics, but the real capacity of answering medical questions in a technical manner and with fidelity to medical established guidelines have been matter of discussion. While some papers say that this tool can provide valuable reliable resources for patients ( 2 – 5 ), other studies draw attention to negative points, such as outdated data, inability to understand psycho-social aspects, lack of transparency, accountability and divergent information's with regard to actual medical literature, with a lot of recommendations against this practice ( 6 – 8 ).

Our objective is to answer the question if ChatGPT can provide reliable technical medical information, consonant with actual medical literature, about phimosis in children for physicians, parents and patients.

Four questions usually made for parents and patients in pediatric urology offices about phimosis were asked to ChatGPT and the answers were compared with the European Association of Urology 2023 recommendation panel guidelines, Pediatric Urology section ( 2 ). The answers and guidelines recommendations were analyzed, compared, reviewed and commented by Pediatric Urology Staff of a University Hospital, considered experts in this issue.

## QUESTIONS

### Question 1: Is topical corticosteroid treatment effective for phimosis?

**ChatGPT:** Topical corticosteroid treatment can be effective for certain cases of phimosis, particularly when the condition is caused by inflammation or scarring. This type of treatment is often considered before more invasive options like circumcision, especially in cases where the phimosis is not causing significant problems and conservative measures are preferred.

**EAU:** Conservative treatment of phimosis with topical corticosteroids (ointment or cream) has a high success rate, but surgical treatment may be considered if preferred by caregivers or patients (Level of Evidence 1b). Offer topical corticosteroids (ointment or cream) as first-line treatment in symptomatic phimosis. (Strong rating)

**Comments:** EAU recommends corticosteroid as a first line treatment, with high success rate, for all patients with phimosis. ChatGPT recommends only for specific cases, and If there is no local complications.

### Question 2: Should Balanitis Xerotica Obliterans (BXO) be treated with circumcision in children?

**ChatGPT** : The management of BXO in children often involves a stepwise approach, and circumcision may be considered in certain cases. However, it's important to note that not all cases of BXO automatically require circumcision. The Treatment options for BXO includes: Topical Corticosteroids, Dilation and Circumcision in cases where conservative measures are not successful.

**EAU:** Offer circumcision in case of BXO (strong level of recommendation).

**Comments:** Here are conflicting information. EAU guidelines recommends offer only surgical treatment for these patients without other options, while ChatGPT considers using corticosteroids and preputial dilation.

### Question 3: Should we perform circumcision for first time episode of paraphimosis?

**ChatGPT:** If manual reduction is unsuccessful or if there are recurrent episodes of paraphimosis, a healthcare provider may explore various treatment options. Circumcision may be considered as a preventive measure in some cases, especially if paraphimosis is a recurring problem or if there are underlying conditions contributing to the issue.

**EAU:** Treat paraphimosis by manual reposition and proceed to surgery if this fails. (Strong level of Recommendation)

**Comments:** Here there is an agreement between both sources. First-line therapy is manual reposition and surgery is only indicated if there is failure. No regards on EAU recommendations are made for recurrent episodes, while ChatGPT recommends surgical treatment in these cases.

### Question 4: What are the main complications of Circumcision?

**ChatGPT:** Bleeding, Infection, Pain and Discomfort, Swelling and Bruising, Irritation or Injury to the Glans, Inadequate or Excessive skin removal, Complications with anesthesia.

**EAU:** Complications following circumcision vary and have been reported to be between 0-30%. Non-healing wounds, hemorrhage, wound infection, meatal stenosis, redundant skin, non-satisfying cosmetic appearance and trapped penis may all occur.

**Comments** : Here there is an almost complete agreement between the two sources, although EAU guidelines provide more detailed information.

## DISCUSSION

Actually, internet is the main source of information for physicians, parents and patients. It can provides technical information for physicians and non-technical for non-healthcare providers. A lot of scientifical effort have been made to explain if ChatGPT can answer technical questions and helps ( 9 , 10 ) in clinical decision-making process at different areas, such as dermatology ( 11 ), surgery ( 12 ), emergency medicine ( 13 ), urology ( 14 ) and other medical areas.

ChatGPT answered questions 1 and 2 differently from classical medical guidelines recommendation, especially in question 2, when formal guidelines recommends surgical treatment, not recommended by the first and it can cause potential damage for the patient if the information provided is used alone. For question 3, there is a partial agreement between the data sources and circumcision is not a formal guideline recommendation after first episode of paraphimosis. In question 4, it appears to have a lot of concordance between both sources, albeit some guideline important topics are not mentioned. In this case both sources of information can be used in a complementary way.

Although ChatGPT can offer good information for general non-healthcare public about the topic "phimosis", there are important conflicts between EAU guidelines and ChatGPT recommendations, especially in guidelines stablished conducts that can lead to wrong medical decision making and potential damage for patients. We do not recommend use of ChatGPT as a technical source of study for physicians and medical decision about phimosis.
